# *PTEN* Hamartoma Tumor Syndrome: Skin Manifestations and Insights Into Their Molecular Pathogenesis

**DOI:** 10.3389/fmed.2021.688105

**Published:** 2021-07-27

**Authors:** Giovanni Innella, Elena Bonora, Iria Neri, Annalucia Virdi, Alba Guglielmo, Laura Maria Pradella, Claudio Ceccarelli, Laura Benedetta Amato, Anna Lanzoni, Sara Miccoli, Giuseppe Gasparre, Roberta Zuntini, Daniela Turchetti

**Affiliations:** ^1^Department of Medical and Surgical Sciences, Center for Studies on Hereditary Cancer, University of Bologna, Bologna, Italy; ^2^Unit of Medical Genetics, IRCCS (Istituto di Ricovero e Cura a Carattere Scientifico) Azienda Ospedaliero-Universitaria di Bologna, Bologna, Italy; ^3^Unit of Dermatology, IRCCS (Istituto di Ricovero e Cura a Carattere Scientifico) Azienda Ospedaliero-Universitaria di Bologna, Bologna, Italy; ^4^Department of Experimental, Diagnostic and Specialty Medicine, Alma Mater Studiorum University of Bologna, Bologna, Italy; ^5^Unit of Dermatology, Ospedale Bellaria-Maggiore di Bologna, Bologna, Italy; ^6^Center for Applied Biomedical Research (CRBA), Bologna, Italy

**Keywords:** *PTEN* gene, phosphatase and tensin homolog deleted on chromosome 10, PHTS, skin lesions, cancer risk, clinical management

## Abstract

Germline *PTEN* pathogenic variants cause a spectrum of disorders collectively labeled *PTEN* Hamartoma Tumor Syndrome (PHTS) and featured by hamartomas, developmental anomalies and increased cancer risk. Studies on experimental models provided evidence that *PTEN* is a “haploinsufficient” tumor-suppressor gene, however, mechanisms involved in the pathogenesis of clinical manifestations in PHTS patients remain elusive. Beyond analyzing clinical and molecular features of a series of 20 Italian PHTS patients, we performed molecular investigations to explore the mechanisms involved in the pathogenesis of *PTEN*-associated manifestations, with special focus on mucocutaneous manifestations. Typical mucocutaneous features were present in all patients assessed, confirming that these are the most important clue to the diagnosis. The most frequent were papules located in the trunk or extremities (73.7%), oral mucosa papules (68.4%), acral/palmoplantar keratosis and facial papules (both 57.9%), according with literature data. Molecular analyses on one trichilemmoma suggested that the wild-type *PTEN* allele was retained and expressed, reinforcing the evidence that *PTEN* does not require a second somatic hit to initiate pathogenic processes. Unexpectedly, one patient also displayed a cutaneous phenotype consistent with atypical mole/melanoma syndrome; no variants were detected in known melanoma genes, but Whole Exome Sequencing showed the rare truncating variant c.495G>A in the *CDH13* gene that might have cooperated with *PTEN*-haploinsufficiency to generate such phenotype. Our findings confirm the reproducibility of known PHTS manifestations in real-world practice, highlighting the role of mucocutaneous manifestations in facilitating prompt diagnosis of the syndrome, and provide some insights into the pathogenic process induced by *PTEN* alterations, which may contribute to its understanding.

## Introduction

*PTEN* Hamartoma Tumor Syndrome (PHTS) defines a spectrum of multisystem disorders caused by *PTEN* gene alterations that are mainly characterized by multiple hamartomas and predisposition to malignant tumor development (1). PHTS includes Cowden syndrome (CS), Bannayan-Riley-Ruvalcaba syndrome (BRRS), *PTEN*-related Proteus syndrome (PS) and Proteus-like syndrome (2). While the latter two are caused by mosaic mutations and therefore clinical manifestations depend on distribution in different tissues (3), CS and BRRS are caused by germline pathogenic variants and their clinical diagnosis is based on specific criteria (4, 5).

More detailed, CS is characterized by multiple hamartomas, high risk of benign and malignant tumors (mainly affecting the thyroid, breast and endometrium), macrocephaly, gastrointestinal polyps, Lhermitte-Duclos disease (LDD) and mucocutaneous manifestations (6). BRRS is a congenital condition characterized by macrocephaly, intestinal hamartomatous polyposis, lipomas and pigmented macules of the glans penis (7).

Typical skin lesions, which in most cases occur by the age of forty, appear as smooth or dyskeratotic papules mainly located in face (especially in perioral area, nose, and eyelids) and extremities (palm-plantar surfaces), and include trichilemmomas, acral keratosis, and papillomatous papules (2, 8). Trichilemmomas are benign tumors of the hair follicle sheath; papillomatous papules are benign cutaneous lesions that are more often localized in areas subjected to pressure (9, 10); acral keratoses consist of foci of keratosis that clinically appear as pits or as plaques/papules (11). Other possible integuments manifestations include lipomas and less frequently café-au-lait spots, hemangiomas, xanthomas, vitiligo, perioral and acral freckles, and acanthosis nigricans. Mucosal involvement is present in over 80% of cases and is mainly affecting the buccal and gingival mucosa, where the lesions can converge giving the oral mucosa a characteristic “cobbled” appearance (12).

The *PTEN* gene encodes a negative regulator of the *PI3K*/*Akt*/*mTOR* pathway and is one of the most frequently mutated genes in cancer (13). Even though the biological roles of this gene have been extensively studied, the link between the dysfunction caused by germline variants and the clinical manifestations remains largely elusive. It is known that although *PTEN* is considered a tumor-suppressor gene, its behavior differs from that of classical tumor-suppressor genes, which act in accordance with Knudson's “two-hit” hypothesis (14, 15). Indeed, evidence showing that the loss of a single *PTEN* allele is enough to promote cancer development has led to the hypothesis of a haploinsufficiency mechanism for this gene (16). It has also been hypothesized that slight reductions in the PTEN dose are sufficient to promote tumors development (17) and that there is a specific tissue sensitivity for the reduction of PTEN levels (18). However, such evidence is almost exclusively based on data on experimental models, with very limited data on human patients (19, 20).

In order to understand if PHTS features detected in routine care are consistent with those reported in the literature and to gain insights into their molecular mechanisms, we characterized a single-center series of Italian patients and performed molecular assessment to explore the mechanisms involved in the pathogenesis of *PTEN*-associated manifestations, with special focus on mucocutaneous manifestations.

## Materials and Methods

### Patients

From 2002 to 2020, *PTEN* gene testing has been undertaken in 101 patients, among those referred to the Cancer Genetics Clinic in Bologna, who displayed clinical features consistent with *PTEN*-associated disease. Once the suspicion of PHTS was raised by clinical genetic assessment, according to International Cowden Consortium operational diagnostic criteria (1, 21) informed consent for *PTEN* gene testing was collected, and a venous blood sample drawn, in the context of a pre-test genetic counseling session. After molecular analysis, a post-test counseling session was arranged to communicate test result and extend the assessment to relatives, whenever appropriate.

### *PTEN* analysis

Genomic DNA was isolated from peripheral blood-EDTA using the QIAmp DNA blood Mini Kit according the manufacturer's protocol (Qiagen, Hilden, Germany). From 2002 to June 2019, sequencing of all 9 exons of the gene, as well as the splice-junctions and the gene promoter region, was performed through bidirectional Sanger sequencing in the Applied Biosystem 3730 DNA Analiyzer automatic sequencer. Since July 2019 the entire *PTEN* coding sequence was included in an NGS panel comprising 19 genes related to cancer predisposition (ThermoFisher Scientific). Overall, 85 patients underwent Sanger sequencing analysis and 16 the NGS panel analysis. In patients in whom no sequence variants of the gene were identified, search for *PTEN* deletion/duplication by Multiplex Ligation-Dependent Probe Amplification (MLPA) was performed, using SALSA MLPA KIT P225-B2 *PTEN* kit and adopting the protocol indicated by the supplier company (MRC Holland). In relatives of index cases, and in tumor tissue samples, the targeted search for known alterations was performed through Sanger sequencing using specifically designed primers and the Applied Biosystem 3730 DNA Analyzer automatic sequencer, according to standard protocols. DNA was extracted from Formalin Fixed Paraffin Embedded (FFPE) samples using QIAamp DNA Mini Kit (Qiagen) according to manufacturer's instructions.

### Clinical Characterization of Patients Carrying *PTEN* Pathogenic Variants

Besides undergoing a careful physical examination during clinical genetic assessment, patients carrying *PTEN* pathogenic variants underwent a complete dermatological evaluation at the clinic for rare skin diseases of the Dermatology Unit of our Hospital. Additional clinical and pathological data were collected from medical records and pathology reports. All clinical data collected during the first evaluation together with those emerged during follow-up examinations were included in a dedicated database. In addition to 18 patients who had performed both clinical assessment and genetic testing at our center, the database also included one patient who was tested at our laboratory after being seen at an external clinic, and one patient who came to our clinic for clinical genetic assessment after having been tested at another laboratory.

### Whole Exome Sequencing

Whole Exome Sequencing (WES) was performed in the patient who presented with multiple melanomas/dysplastic nevus in addition to features suggestive of PHTS and his parents (trio analysis); 100 ng of genomic DNA extracted from peripheral blood were tagmented according to Nextera® Rapid Capture Enrichment protocol. The fragmentation was verified using the Agilent Bioanalyzer and library enrichment was performed by hybridization using the coding exome exon kit (Nextera) and sequenced with Illumina HiScan SQ platform at 100 bp paired-ends. Reads were aligned with BWA to the reference genome (hg19). Aligned reads were treated for realignment and base quality score recalibration with GATK, and for duplicate removal with PicardTools (http://picartools.sourceforge.net). Alignment statistics were collected with SAMtools and GATK. Coverage statistics over the targeted regions were calculated with GATK. Variant calling and filtering by quality was performed by GATK. Variants passing quality filters were annotated separately against NCBI RefGene (http://www.ncbi.nlm.nih.gov) and UCSC KnownGene (http://genome.ucsc.edu).

### *PTEN* Transcription Analysis

Total RNA was extracted from a verrucous skin lesion of patient Ia using Trizol Reagent (Invitrogen) according to the manufacturer's method. A total of 1 μg RNA was reverse-transcribed into cDNA using Oligo-dT primers and GoScript Reverse Transcription System (Promega) according to the supplier's protocol. In order to verify the expression of both alleles, the PCR product obtained using primers designed on cDNA was cloned into pcR2.1-TOPO vectors using the TOPO TA Cloning Kit and related protocol (Invitrogen, San Diego, CA, USA). DH5α strain *E. coli* cells (Invitrogen, San Diego, CA, USA) were employed as a host for transformation, and colonies containing the recombinant plasmids were screened. The PCR products were purified with QIAquick PCR purification kit (Qiagen) and directly sequenced using an ABI PRISM 3730 automated DNA sequencer and a Big Dye Terminator DNA sequencing kit (Applied Biosystem, Foster City, CA, USA).

### Microsatellite Analysis

Microsatellite analysis with markers D16S3091 and D16S520 at chromosome 16q23.3, where the *CDH13* gene maps, was performed using 40 ng of total DNA extracted form peripheral blood or thyroid tumor tissue in the following PCR conditions: 15 μl final volume, KAPA Fast MasterMix (Sigma-Aldrich, St. Louis, USA), 0.5 μM primers, T_a_ = 56°C 40 cycles, as previously described (22). PCR products were run onto ABI3730 sequencing machine and analyzed with GeneMapper™ Software v.4.0 (Applied Biosystems).

### Immunohistochemistry

Immunostaining was performed on 2 μm thick sections, from paraffin-embedded, formalin-fixed tissue blocks of breast cancer derived sample of the patient mounted on positively charged glass. They were incubated with Phospho-AKT-Ser473 Rabbit primary antibody (Cell Signaling Technology, Inc. Danvers, MA) and PTEN Rabbit primary antibody (Zymed Laboratories, Invitrogen, Paisley, UK).

## Results

### Clinical Manifestations in PHTS Patients

Twenty patients belonging to 14 unrelated families carry *PTEN* variants considered as causative according to criteria by Mester et al. (23) and are therefore conclusively diagnosed with PHTS. These include four children aged between one and 12 years (mean age: 6.3 years) at diagnosis, and 16 adults with ages ranging from 27 to 77 years (mean age: 43.7 years) at diagnosis. Clinical features and *PTEN* variants of patients are reported in Table 1.

**Table 1:** Clinical and molecular features of PHTS patients described in this study.

**Family**	**Patient ID**	**Age^a^**	**Sex**	**LDD**	**MM**	**Mac^b^**	**BC**	**ETC**	**EC**	**BTD**	**DD/ASD**	**GI Pol**	**FBC**	**Ut Fib/Lei**	**OBL**	**GUC**	**OC**	**FH^c^**	**Germline *PTEN* variant^d^ (rule code^e^)**
B-24-06	Ia	47	M	N	Y	Y	N	N	–	Y	N	Y	N	–	N	N	N	Y	c.306del; p.Lys102Asnfs*11 (PVS1)
B-24-06	Ib	63	F	N	Y	Y	Y	N	N	N	N	Y	N	Y	N	N	Y	Y	c.306del; p.Lys102Asnfs*11 (PVS1)
43-O-07	II	39	M	Y	Y	Y	N	Y	–	N	N	Y	N	–	N	N	N	N	Whole gene deletion (PVS1)
7-O-08	III	44	M	Y	Y	Y	N	N	–	Y	N	Y	N	–	N	N	N	N	c.618del; p.Phe206Leufs*15 (PVS1)
35-O-08	IV	39	F	N	Y	Y	Y	N	N	Y	N	NA	N	Y	N	N	N	N	c.71A>T; p.Asp24Val (PS3_P)
30-O-09	V	12	M	N	Y	Y	N	N	–	N	Y	NA	N	-	N	N	N	N	Intragenic deletion of exon 2 (PVS1)
14-O-10	VIa	43	F	N	Y	Y	Y	N	N	Y	N	Y	N	Y	N	N	N	Y	c.884dup; p.Cys296Metfs*2 (PVS1)
14-O-10	VIb	38	M	N	Y	App	N	N	–	Y	N	NA	N	–	N	N	N	Y	c.884dup; p.Cys296Metfs*2 (PVS1)
14-O-10	VIc	36	M	N	Y	App	N	Y	–	N	N	Y	N	–	N	N	N	Y	c.884dup; p.Cys296Metfs*2 (PVS1)
26-O-10	VII	39	M	N	Y	Y	N	Y	–	Y	N	Y	N	–	N	Y	Y	N	c.1026+1G>A; p.? (PVS1)
45-O-12	VIII	37	M	N	Y	Y	N	N	–	Y	N	NA	N	–	N	N	N	Y	c.1003C>T; p.Arg335* (PVS1)
14-PTD-12	IXa	38	F	N	Y	Y	Y	Y	N	N	N	Y	Y	N	N	N	N	Y	c.253+1G>A; p.? (PVS1)
14-PTD-12	IXb	4	F	N	Y	Y	N	N	N	N	N	NA	N	N	N	N	N	Y	c.253+1G>A; p.? (PVS1)
16-O-13	X	27	M	N	Y	Y	N	N	–	Y	N	Y	N	–	N	N	N	Y	c.325dup; p.Asp109Glyfs*6 (PVS1)
32-O-13	XIa	43	M	N	Y	Y	Y	N	–	Y	N	Y	N	–	N	N	N	Y	c.1003C>T; p.Arg335Ter (PVS1)
32-O-13	XIb	44	F	N	Y	Y	N	N	N	Y	N	NA	N	N	N	N	N	Y	c.1003C>T; p.Arg335Ter (PVS1)
95-M-15	XII	8	M	N	Y	Y	N	N	–	N	N	NA	N	–	N	N	N	N	c.800dup; p.Asp268Glyfs*30 (PVS1)
214-O-15	XIIIa	1	M	N	Y	Y	N	N	–	N	Y	NA	N	–	N	N	N	Y	c.264T>A;p.Tyr88* (PVS1)
214-O-15	XIIIb	29	M	N	Y	Y	N	N	–	Y	N	N	N	–	N	N	N	Y	c.264T>A;p.Tyr88* (PVS1)
38-OB-19	XIV	77	F	N	N	NA	Y	N	Y	Y	N	N	N	N	Y	N	Y	N	c.801+1G>T; p.? (PVS1)

a *At first assessment*.

b *Occipital frontal circumference ≥ 97th percentile*.

c *For PHTS manifestations or PTEN variants*.

d *NM_000314.8*.

e *Rule code to defining pathogenicity according to Mester et al. (23)*.

*LDD, Lhermitte-Duclos Disease; MM, Mucocutaneous Manifestations; Mac, Macrocephaly; BC, Breast Cancer; ETC, Epithelial Thyroid Cancer; EC, Endometrial Carcinoma; BTD, Benign Thyroid Disease; DD, Developmental Delay; ASD, Autism Spectrum Disorder; GI Pol, GastroIntestinal Polyposis; FBC, Fibrocystic Breast Disease; Ut Fib/Lei, Uterine Fibroids/Leiomyomas; OBL, Other Benign Lesions; GUC, GenitoUrinary Cancer; OC, Other cancer; FH, Family History; M, Male; F, Female; Y, Yes; N, No; App, Apparent; NA, Not available*.

*In RED features that led to genetic assessment*.

Reasons that led to genetic assessment and subsequently to the diagnosis of PHTS in index cases were variable: presence of mucocutaneous lesions in five cases, occurrence of multiple/juvenile/familial neoplasms in three cases, finding of LDD in two cases, association of macrocrania and developmental delay (DD)/autism spectrum disorder (ASD) in two cases, and detection of gastrointestinal polyposis in two cases.

As shown in Table 1, all patients in whom head circumference was measured had macrocephaly (17 of 17, 100%), and all those in whom careful physical examination was performed had typical mucocutaneous lesions (19 of 19, 100%); in addition, 5 out of 6 adult women had been affected by invasive breast cancer (83.3%). Other common manifestations were gastrointestinal polyposis (10/12 of those in which it was investigated, 83.3%), benign thyroid diseases (12/16 adult subjects, 75%) and uterine fibroids/leiomyomas (3/6 adult women, 50%). Epithelial thyroid carcinomas were present in 4/16 of adult subjects (25%). Less common findings were endometrial carcinoma (1/6 adult women, 16.67%), fibrocystic breast disease (1/6 adult women, 16.67%), LDD (2/16 adults, 12.5%), DD/ASD (2/20 patients, 10%), genitourinary cancer (1/16 adults, 6.25%) and other benign lesions (1/20 patients, 5%). Five patients (25%) declined follow-up after diagnosis, while the other 15 (75%) were followed up for an average time of 4.9 years after first assessment. Frequency of each clinical feature in this series is shown in Table 2.

**Table 2:** Frequencies of clinical features in our series.

**Feature**	**Number^a^**	**Total^b^**	**%**
LDD	2	16	12.5
Mucocutaneous manifestations	19	19	100
Macrocephaly^c^	17	17	100
Invasive breast cancer	5	6	83.3
Epithelial thyroid cancer	4	16	25
Endometrial carcinoma	1	6	16.67
Benign thyroid disease	12	16	75
DD/ASD	2	20	10
GI polyposis	10	12	83.3
Fibrocystic breast disease	1	6	16.67
Uterine fibroids/leiomyomas	3	6	50
Other benign lesions	1	20	5
Genitourinary cancer	1	16	6.25

a *Of subjects with the diagnosed feature*.

b *Of subjects in which the feature was investigated (for cancers and thyroid disease only adult patients are considered, for breast and endometrial diseases only adult women)*.

c *Confirmed by measurement*.

*LDD, Lhermitte-Duclos Disease; DD, Developmental Delay; ASD, Autism Spectrum Disorder; GI, GastroIntestinal*.

### Features of Cancers Arisen in PTHS Patients

Cancer histories of patients in our series are summarized in Table 3. All adult women developed invasive breast cancer at a mean age of 47.2 years, except one (patient IXa), who underwent bilateral mastectomy for severe fibrocystic disease at the age of 26, with lobular carcinoma *in situ* (LCIS) found at pathology examination; of the five patients with invasive breast cancer, two developed metachronous contralateral breast cancer 2.1 years after the first diagnosis, on average. Among adult patients, four (25%) developed epithelial thyroid carcinomas at a mean age of 34.3 years, in three cases it was of follicular type, while the fourth patient had multifocal papillary carcinoma and oxyphilic cell carcinoma. Endometrial cancer was diagnosed in 1 out of 6 adult women, at the age of 63 years, while 1 of 16 adults developed genitourinary cancer (papillary renal carcinoma) at the age of 49 years. Cancer types less commonly reported in PHTS patients arose in individual patients in this series: colon adenocarcinoma (diagnosed at 61 years of age), multiple melanomas (first diagnosed at 26 years of age), lung carcinoid and gastric adenocarcinoma (both diagnosed in the same patient at 76 years of age).

**Table 3:** Tumors in our patients.

**Patient**	**1st tumor (age)**	**2nd tumor (age)**	**3rd tumor (age)**	**4th tumor (age)**	**5th tumor (age)**	**6th tumor (age)**
Ib	Breast cancer (51)	Colon adenocarcinoma (61)				
II	Papillary thyroid carcinoma (39)	Thyroid oxyphilic cell carcinoma (39)	LDD (43)			
III	LDD (43)					
IV	Breast cancer (32)	Breast cancer (36)				
VIa	Breast cancer (41)					
VIc	Follicular thyroid carcinoma (34)					
VII	Multiple menalomas (from 26)	Follicular thyroid carcinoma (38)	Papillary renal carcinoma (49)			
IXa	Follicular thyroid carcinoma (26)	Breast LCIS (26)				
XIa	Breast cancer (40)	Breast cancer (40)				
XIV	Breast DCIS (42)	Breast LCIS (44)	Endometrial cancer (63)	Breast Cancer (72)	Lung carcinoid (76)	Gastric tubular adenocarcinoma (76)

*LDD, Lhermitte-Duclos disease; LCIS, Lobular Carcinoma in situ; DCIS, Ductal Carcinoma in situ*.

Overall, 9/16 adult patients (52.3%) developed at least one malignancy at a mean age of 33 years at first diagnosis, and four developed multiple primary cancers: patient Ib breast cancer at 51 years and colon adenocarcinoma at 61 years; patient VII melanoma at 26 years, follicular thyroid carcinoma at 38 years and papillary renal carcinoma at 49 years; patient IXb breast LCIS and follicular thyroid carcinoma both at 26 years of age; patient XIV breast ductal carcinoma *in situ* (DCIS) cancer at 42 years, breast LCIS at 44 years, endometrial cancer at 63 years, invasive breast cancer at 72 years and lung carcinoid and gastric adenocarcinoma both at 76 years, and also presented adrenal adenoma and renal angiomyolipomas.

Finally, cerebellar dysplastic gangliocytoma (LDD), a pathognomonic sign of CS, was diagnosed in 2/16 patients (12.5%) at a mean age of 43 years, but a third patient (patient X) reported having undergone neurosurgery for removal of an unspecified cerebellar mass when he was 8 years old and stated that the mother had died around 40 years of age due to an unknown cerebellar disease (clinical records were unavailable).

### Mucocutaneous Manifestations

Nineteen patients underwent careful skin assessment by expert dermatologists: all presented with one or more mucocutaneous lesions.

The most frequent manifestations were papules located in the trunk or extremities, present in 14/19 patients (73.7%), followed by oral mucosa papules, present in 13/19 patients (68.4%), acral/palmoplantar keratosis and facial flesh-colored non-keratotic papules, both present in 11/19 patients (57.9%); a true scrotal tongue was identified in 3/19 patients (15.8%); lesions removed from three patients were histologically diagnosed as trichilemmomas, which appeared as skin colored to yellow-brown, smooth papules usually measuring 1-to-5 mm on the central face. Oral papillomatosis or papules are few millimeters white-grayish soft papules usually coalescing in cobblestone pattern in upper and lower gums and in two cases in tongue (IXa and IXb). Acral keratoses involved limbs as papular lesions, 2–4 mm in diameter with a keratotic surface, and palm and soles as multiple bilateral small keratotic papules with a central depression (pits). In two cases keratotic papules were detected also in ear pavilions and retroauricolar area (IXa and XIIIb).

Other manifestations found in multiple patients were vascular anomalies (particularly angiomas/hemangiomas), present in 6/19 patients (31.6%), cutaneous lipomas and fibroids, both present in 3/19 patients (15.8%), café-au-lait spots, present in 3/19 patients (15.8%), nail anomalies (including trachyonichia and absent lunula) and hamartomas, both found in 2/19 patients (10.5%). Patient IV developed an atypical vascular proliferation on the right breast, after radiation for cancer.

Neurofibromas, keratoacanthomas, melanomas and epitheliomas were all found in individual subjects (5.3%). Cutaneous neuromas were observed in one patient (XII) as dome-shaped translucent multiple skin-colored papules of the hand, and at histological examination pathologists confirmed the neural nature although it was misdiagnosed as plexiform neurofibroma.

**Figure 1:**
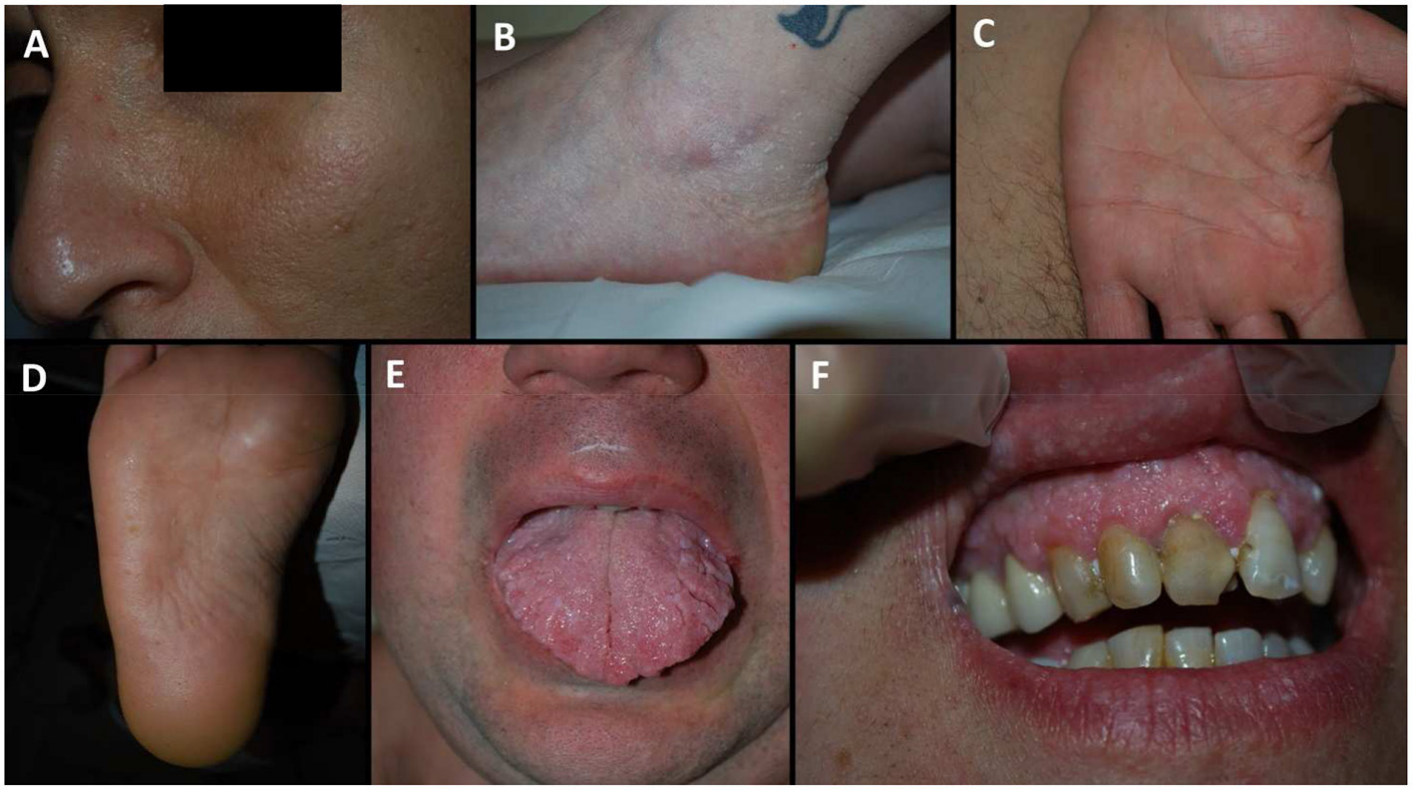
Dermatological manifestation of our patients. **(A)** patient IXa, facial papules; **(B)** patient IV, acral keratotic papules on the foot; **(C)** patient XIb, pits on the palm; **(D)** patient VIc, pits on the sole; **(E)** patient VIc, scrotal tongue; **(F)** patient IXb, oral papules of the gums arranged in a cobblestone pattern.

**Figure 2:**
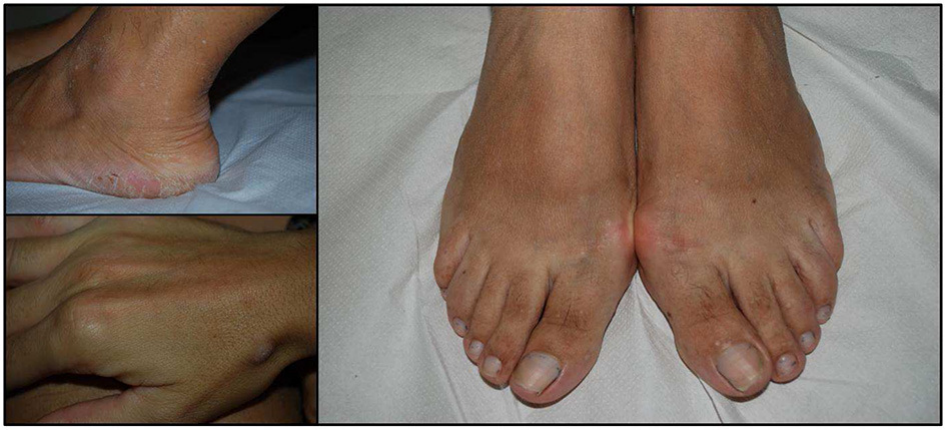
Acral keratosis in three cases. The number of lesions is variable and may involve hands and feet but also the distal part of legs. They appeared as papular lesions, 2–4 mm in diameter with a keratotic surface.

Finally, pigmented macules on the glans penis, a hallmark of BRRS patients, were identified only in two children evaluated for macrocrania (XII and XIIIa) and intellectual disability (patient XIIIa). Mucocutaneous manifestations of each patient are summarized in Table 4, and exemplar pictures are shown in Figures 1, 2. Regarding the four children reported in this series, all presented with at least one typical mucocutaneous manifestation at early age: in patient V, hyperkeratosis of the palms and hypopigmented lesions were detected at the age of 8 years; patient IXb had a warty lesion from birth; in patient XII, cutaneous lipomas and papular lesions appeared at the age of 3 years; in patient XIIIa, penile freckling was noticed in the first year of life.

**Table 4:** Mucocutaneous manifestations in our series.

**Patient feature**	**Ia**	**Ib**	**II**	**III**	**IV**	**V**	**VIa**	**VIb**	**VIc**	**VII**	**VIII**	**IXa**	**IXb**	**X**	**XIa**	**XIb**	**XII**	**XIIIa**	**XIIIb**	**Total (%)**
Trichilemmomas^a^																				3 (15.8)
Acral/palmoplantar keratosis																				11 (57.9)
Facial papules/papillomas																				11 (57.9)
Trunk/extremities papules/papillomas																				14 (73.7)
Oral papules/papillomas																				13 (68.4)
Scrotal tongue																				3 (15.8)
Penile freckling																				2 (10.3)
Vascular anomalies^b^																				6 (31.6)
Skin lipomas																				3 (15.8)
Skin fibromas																				3 (15.8)
Café-au-lait spots																				3 (15.8)
Neuromas/neurofibromas																				1 (5.3)
Melanomas																				1 (5.3)
Hamartomas																				2 (10.5)
Epitheliomas																				1 (5.3)
Keratoacanthomas																				1 (5.3)
Nail anomalies^c^																				2 (10.5)

a *Histologically confirmed*.

b *Including angiomas, hemangiomas, arteriovenous malformations*.

c *Including trachyonichia and absent lunula*.

*To facilitate understanding, each feature has been associated to a specific color*.

Patient VII presented with a complex cutaneous phenotype comprised of both features of PHTS and of inherited melanoma syndrome. Indeed, starting at the age of 26 years, the patient underwent the removal of more than 60 pigmented skin lesions, many of which were diagnosed as dysplastic nevi or melanomas (mostly *in situ*) on histopathological examination. He was first referred for hereditary melanoma assessment, but the constitutional analysis of major melanoma genes (*CDKN2A* and *CDK4* sequence analysis and *CDKN2A* MLPA analysis) failed to find any significant variant. As the personal history of early-onset thyroid disease (hemithyroidectomy for goiter with follicular adenoma at age 19, followed by follicular carcinoma at the age of 38 years), together with the presence of macrocephaly and consistent skin lesions (several flat verrucous lesions and keratoderma of the hands), was suspicious for PHTS, *PTEN* testing was undertaken and led to the molecular confirmation of PHTS. During follow-up, he underwent removal of additional skin lesions (one hemangioma, one oral verrucous papula, other dysplastic nevi) and gastrointestinal polyps, and was diagnosed with papillary renal carcinoma at the age of 49 years.

### Molecular Characterization

Thirteen different germline *PTEN* pathogenic variants were detected in 20 patients (11 by gene sequence analysis and two by MLPA analysis), as reported in Table 1. Frameshift variants accounted for 38.5% (5/13), splice-site variants for 23.1% (3/13), nonsense variants and deletions for 15.4% each (2/13), and missense variants for 7.7% (1/13). The only missense variant, c.71A>T; p.Asp24Val (patient IV), was demonstrated to be deleterious by functional analyses (24). Regarding the deletions, one was intragenic and limited to exon 2 (patient V), while the other (patient II) encompassed the entire *PTEN* sequence and other genes, as previously described (25). Among sequence variants, 3/11 (27.3%) were located in exon 5, which encodes the phosphate core motif and in which ~40% of *PTEN* variants are located (26), while the others were scattered along the remaining gene sequence.

In order to gain insights into the molecular pathogenesis of skin lesions associated with PHTS, we performed molecular characterization of a trichilemmoma removed from the labial commissure of patient Ia. First, both the alleles (the wild-type and the one carrying the c.306del variant) were demonstrated to be transcribed through retro-transcription and cDNA sequencing, and preferential expression of one over the other was excluded by cloning in bacteria and sequencing of individual colonies, with the wild-type allele detected in 11 out of 21 colonies (52%).

**Figure 3:**
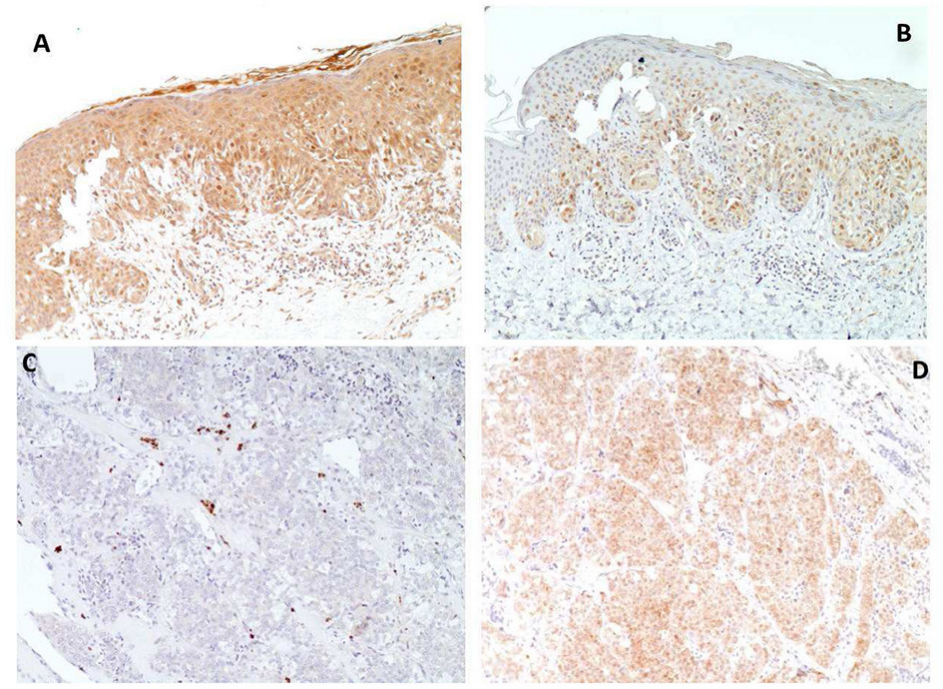
Immunohistochemical analyses of melanoma **(A,B)** and thyroid carcinoma **(C,D)** of patient VII. PTEN immunostaining shows strong nuclear and cytoplasmic immunoreactivity in melanoma **(A)** and absent reaction in thyroid carcinoma **(C)**, conversely Phospho-AKT-Ser473 immunostaining shows weak reaction in melanoma **(B)**, compared to intense staining in thyroid carcinoma **(D)** (Magnification X 100).

In patient VII, the detection of the pathogenic variant c.1026+1G>A in *PTEN*, which was demonstrated to be *de novo* by segregation analysis, could explain the clinical manifestations of PHTS. Indeed, the variant has been shown to cause retention of 190 nucleotides from intron 8 (27) and is classified as pathogenic (ClinVar VCV000183722.4). However, the occurrence of numerous melanomas and nevi could not be conclusively justified by the *PTEN* variant. In order to explore potential links between the *PTEN* variant and melanoma development, we analyzed the status of the variant c.1026+1G>A and the expression of PTEN and phosphorylated-AKT in melanoma, as well as in thyroid cancer tissue. In the melanoma, both the wild-type and the variant *PTEN* alleles were detected by Sanger sequencing, the PTEN protein was shown to be expressed by immunohistochemistry, and staining for phospho-AKT was weak; conversely, in the thyroid carcinoma only the variant allele was detected, supporting LOH; consistently, IHC analysis of PTEN was negative, whereas there was an intense staining for phospho-AKT (Figure 3). In the attempt to identify additional genetic variants that could explain the multiple melanoma phenotype, WES was performed: first, an *in silico* panel of genes involved in predisposition to melanoma (*CDKN2A, CDK4, BAP1, POT1, MITF, MC1R, TERT, XRCC3*) was analyzed, with no pathogenic variants were found. Then, filtering for novel/rare pathogenic variants (i.e., stop codon, frameshift, non-conservative missense changes, splice-site alteration) led to the detection of the variant c.495G>A in *CDH13*, predicted to generate a premature stop at codon 165 (p.Trp165Ter). Segregation analysis in the family showed that the *CDH13* variant was inherited from the asymptomatic mother. Microsatellite analysis led to detection of loss of heterozygosity at the CDH13 locus in thyroid carcinoma, while in the melanoma tissue the c.495G>A variant was in heterozygous state.

## Discussion

Patients with PHTS face an increased risk of cancer, particularly of breast and endometrium in women (lifetime risk 67–85% and 19–28%, respectively) and of epithelial thyroid and renal cells in both genders (lifetime risk 6–38% and 2–24%, respectively) (28, 29). For this reason, early identification of these patients is crucial, as undertaking specific surveillance programs may reduce the risk of developing, or dying from, those malignancies (21, 30). Indeed, although PHTS features often present early in life, a proportion of patients remain undiagnosed until adulthood, after they have already developed malignancies. Yet, in some cases, referral for genetic assessment is motivated by the suspicion of another disease, such as Familial Adenomatous Polyposis (31) or dysplastic nevi/melanoma syndrome (patient VII in this report).

Therefore, recognizing typical mucocutaneous manifestations is crucial to allow patients be properly diagnosed and managed: the presence of three or more of trichilemmomas, acral keratoses, mucocutaneous, neuromas, and oral papillomas, which generally occur by the second or third decade of life, corresponds to one major criteria (32). In this series, all the patients undergoing complete dermatology assessment displayed typical mucocutaneous features (2). Trichilemmomas, benign follicular tumor with outer root sheath differentiation which are regarded to be the most suggestive lesions of PHTS patients (33), were histologically confirmed in 15.8% of our patients; histological assessment is generally required to confirm the diagnosis of trichilemmomas, because they are clinically indistinguishable from trichoepitheliomas, fibrofolliculomas, and other benign skin lesions. Among our patients, 89.5% showed multiple papillomas and 57.9% presented with acral palmoplantar keratoses; according to some authors, these latter lesions, which typically appear as pits with punctate central depression on dorsal aspect of hands and feet and in palmoplantar area, should be considered as the most sensitive and specific clinical finding of PHTS (4, 34). Oral papillomatosis and papules, inflammatory fibroepithelial hyperplasias involving gingiva, lip and all oral cavity, were present in 68.4% of our patients consistently with 14–76% in the literature (2). One patient (5.3%) presented with a plexiform neuroma; indeed, neuromas (hamartomas of the peripheral nerve sheath uncommon in the general population) have been reported in as many as 5–10% of PHTS patients, with childhood onset in more than half cases (35). Concerning the age at the onset of mucocutaneous manifestations, all the adults, included those referred for other features, showed extensive involvement, while in children less numerous yet typical features were detected by careful assessment, even at a very early age. None of the four children present in this series had scrotal tongue, vascular anomalies, skin fibromas, melanomas, epitheliomas, keratoacanthomas or nail anomalies at the time of the dermatological evaluation. These findings are consistent with complete penetrance for skin phenotype at early age, with manifestations increasing by number and heterogeneity over time.

Additional molecular studies were performed in some of our patients, as described here and in previous reports, in the attempt of gaining insights into mechanisms involved in clinical manifestations of PHTS. First, we have found that in the majority of lesions analyzed the wild-type *PTEN* allele is retained (findings summarized in Supplementary Table 1): thyroid papillary and oxyphilic cell carcinomas from patient II (25, 36), breast carcinoma from patient IV (24), colon polyps from patients Ia and IXa (31), labial verruca from patient Ia and melanoma from patient VII (present study). The follicular thyroid carcinoma of patient VII is the only exception: in the DNA extracted from the tumor, in fact, LOH clearly emerges, suggesting that the wild-type allele is at least partially deleted; consistently, the IHC analyses demonstrates absent expression of the PTEN protein associated with overexpression of phosphorylated-AKT. A similar genetic status has been described in the follicular thyroid carcinoma cell line FTC-133 that had one *PTEN* allele deleted and the remaining allele harbored a splice variant, which suggests that in this tumor type biallelic *PTEN* loss may be more frequent (37). In the verrucous lesion of patient Ia, the wild-type allele was also found to be regularly transcribed, with no allelic imbalance clearly detected, and translated, therefore a significant reduction of the PTEN protein is not supported. These results confirm that *PTEN* fails to follow the Knudson two-hit mechanism expected for classical tumor-suppressor genes and support the hypothesis of a haploinsufficiency mechanism for this gene (15, 16). To the best of our knowledge, evidence supporting this hypothesis are mainly based on experimental models (17–20), and therefore the verification on real patients, as performed in this and in our previous works, is fundamental to better understand the pathogenic mechanism of *PTEN*.

Furthermore, the search for somatic mutations suggested evidence for diverse cancer genes being mutated in diverse tumor types: the papillary thyroid carcinoma of patient II harbored the *BRAF* V600E mutation, while the synchronous thyroid oncocytoma displayed a mutation in the mitochondrial DNA and a deletion encompassing the *FLCN* gene (25); in the breast carcinoma of patient IV, pAKT was shown to be overexpressed in spite of the absence of *PTEN* LOH and of common somatic mutations of the pathway (*PI3KCA* and *AKT*), possibly implying rarer mutations (24). It could be hypothesized that the presence of a constitutional *PTEN* mutation and the consequent reduction of the PTEN dosage creates a “permissive” environment to the carcinogenic effect of somatic mutations in the cells; while in the wild-type individual most of the stochastic mutations in oncogenes cause cell senescence (38, 39), in the *PTEN* mutation heterozygotes the same mutations may have higher chances of initiating carcinogenesis.

Patient VII was of particular interest, as the occurrence of multiple atypical nevi and melanomas is unexpected in PHTS patients, who face a cumulative risk of melanoma lower than 6% by the age of 70 years (28, 29) and who have been reported with single melanomas without juvenile onset (29, 40). In addition, IHC analysis of the melanoma of the patient showed that PTEN was normally expressed and phospho-AKT down-regulated, which provides evidence against a direct role of the de-regulation of this pathway in the development of this tumor. No other genes explaining the multiple melanoma phenotype have been detected in the patient, whereas WES led to identify only a rare *CDH13* truncating variant. As demonstrated for other conditions, in particular neurodevelopmental disorders, the presence of additional rare variants in the genetic background of one individual carrying a pathogenic alteration can sometimes explain complex phenotypes and variable expressivity (41, 42). This model could be applied to the case of patient VII: on one hand, the fact that the *CDH13* variant was inherited from the healthy mother excludes a fully-penetrant effect in causing multiple melanomas, but on the other hand, it may be have played a role in the phenotype of the patient, possibly as the result of the co-existence with the *PTEN* variant, as *CDH13* has previously been demonstrated to be implied in melanomagenesis (43–45).

In conclusion, although the limited number and variety of the patients here described do not allow to provide accurate figures of *PTEN*-associated mucocutaneous manifestations, it is highlighted the importance of a careful clinical evaluation for the prompt diagnosis and proper management of patients with PHTS, with particular attention to dermatological, even subtle, manifestations. Furthermore, the molecular investigations carried out on tissues of real patients taken from this case series provide some insights into the mechanisms implicated in the development of clinical manifestations of PHTS, offering additional support to the evidence that *PTEN* does not behave like a classic tumor-suppressor gene and highlighting the importance of the individual genetic background in the expressiveness of this pathology. It is proposed that *PTEN* haploinsufficiency may favor the carcinogenetic effect of “non-specific” somatic mutations and/or interact with germline low-penetrance variants, with the specific gene involved influencing the type of tumor eventually developed.

## Data Availability Statement

The whole exome sequencing datasets generated for this study are publicly available. This data can be found in SRA database with accession number PRJNA720574 at the following link: https://www.ncbi.nlm.nih.gov/sra/PRJNA720574.

## Ethics Statement

The studies involving human participants were reviewed and approved by Ethical Board of Hospital S. Orsola-Malpighi, Bologna, Italy (112/2014/O/Tess). Written informed consent was obtained from the individual(s), and minor(s)' legal guardian/next of kin, for the participation in the study and the publication of any potentially identifiable images or data included in this article.

## Author Contributions

GI and DT: conceptualization, clinical evaluation, data acquisition and analysis, manuscript preparation and revisions. EB, LP, LA, GG, and RZ: molecular analysis and results' interpretation. IN, AV, AG, AL, and SM: clinical evaluation. CC: immunohistochemical analysis. All authors read and approved the final manuscript prior to submission.

## Conflict of Interest

The authors declare that the research was conducted in the absence of any commercial or financial relationships that could be construed as a potential conflict of interest.

## Publisher's Note

All claims expressed in this article are solely those of the authors and do not necessarily represent those of their affiliated organizations, or those of the publisher, the editors and the reviewers. Any product that may be evaluated in this article, or claim that may be made by its manufacturer, is not guaranteed or endorsed by the publisher.
