# Supplementary material for: PTEN Hamartoma Tumor Syndrome: Skin Manifestations and Insights Into Their Molecular Pathogenesis
Source: Front Med (Lausanne). 2021 Jul 27;8:688105. doi: 10.3389/fmed.2021.688105 (PMC8353102; doi:10.3389/fmed.2021.688105)
Supplement: Supplementary file 1 [file Table_1.docx]

**Supplementary Table 1.** Findings supporting the absence of LOH in patients in this series (data previously published and present work).

| **Patient ID** | **Germline *PTEN* variant** | **Main results** | **Reference** |
| --- | --- | --- | --- |
| **II** | Whole gene deletion | IHC in tumors (thyroid papillary and oxyphilic cell carcinomas): PTEN expressed  *PTEN* sequence in tumor tissues: wt | Pradella et al, 2011 (25) |
| **IV** | c.71A>T;p.Asp24Val | *PTEN* sequence in tumor tissue (breast carcinoma): variant at the heterozygous status, no other variants | Pradella et al, 2014 (24) |
| **Ia**/**IXa** | c.306del;p.Lys102Asnfs*11/c.253+1G>A;p.? | Variant at the heterozygous status in polyp tissues of both patients and labial verruca of patient Ia | Innella et al, 2020 (31) |
| **VII** | c.1026+1G>A;p.? | Variant at the heterozygous status in tumor tissue (melanoma) | Present study |

IHC = Immunohistochemistry; wt = wild type.
